# Evaluating Trends in COVID-19 Research Activity in Early 2020: The Creation and Utilization of a Novel Open-Access Database

**DOI:** 10.7759/cureus.9943

**Published:** 2020-08-22

**Authors:** Rebecca C Jones, Jasper C Ho, Hannah Kearney, Meghan Glibbery, Daniel L Levin, John Kim, Sara Markovic, Jillian Howden, Maya Amar, Mark A Crowther

**Affiliations:** 1 Medicine, McMaster University, Hamilton, CAN

**Keywords:** covid-19, research, trends, covidreview, pandemic, coronavirus, sars-cov-2, novel, severe acute respiratory syndrome coronavirus 2

## Abstract

Introduction

The coronavirus disease 2019 (COVID-19) pandemic has been unprecedented in recent history. The rapid global spread has demonstrated how the emergence of a novel pathogen necessitates new information to advise both healthcare systems and policy-makers. The directives for the management of COVID-19 have been limited to infection control measures and treatment of patients, which has left physicians and researchers alone to navigate the massive amount of research being published while searching for evidence-based strategies to care for patients. To tackle this barrier, we launched CovidReview.ca, an open-access, continually updated, online platform that screens available COVID-19 research to determine higher quality publications. This paper uses data from this review process to explore the activity and trends of COVID-19 research worldwide over time, while specifically looking at the types of studies being published.

Materials and Methods

The literature search was conducted on PubMed. Search terms included “COVID-19”, “severe acute respiratory syndrome coronavirus 2”, “coronavirus 19”, “SARS-COV-2”, and “2019-nCoV”. All articles captured by this strategy were reviewed by a minimum of two reviewers and categorized by type of research, relevant medical specialties, and type of publication. Criteria were developed to allow for inclusion or exclusion to the website. Due to the volume of research, only a level 1 (title and abstract) screen was performed.

Results

The time period for the analysis was January 17, 2020, to May 10, 2020. The total number of papers captured by the search criteria was 10,685, of which 2,742 were included on the website and 7,943 were excluded. The greatest increase in the types of studies over the 16 weeks was narrative review/expert opinion papers followed by case series/reports. Meta-analyses, systematic reviews, and randomized controlled trials remained the least published types of studies.

Conclusions

The surge of research that accompanied the COVID-19 pandemic is unparalleled in recent years. From our analysis, it is clear that case reports and narrative reviews were the most widely published, particularly in the earlier days of this pandemic. Continued research that falls higher on the evidence pyramid and is more applicable to clinical settings is warranted.

## Introduction

The global spread and international response to the coronavirus diseases 2019 (COVID-19) pandemic has been unprecedented. After the first cluster of patients identified in December 2019 in Wuhan, China, the burden of disease has rapidly spread, currently affecting more than seven million patients worldwide [1,2]. The emergence of a novel pathogen necessitates a need for information to inform both healthcare systems and policy-makers. Rapidly conducted research and developments in diagnosis, treatment, and prevention have resulted in an onslaught of information that can be difficult to navigate and apply clinically. Further complicating this are unsubstantiated claims by the media, which has led to the spread of misinformation, unfounded fear, and detraction from reliance on high-quality evidence-based research.

The World Health Organization (WHO) and the US Centers for Disease Control and Prevention’s (CDC) directives for the management of COVID-19 have been limited to infection control and symptomatic management of patients [3,4]. This has left frontline physicians navigating the enormous amount of research being published, searching for evidence-based strategies to care for their patients. A significant barrier to effectively utilizing COVID-19 research is the immense volume and rapid publication of studies of variable quality [5,6]. There are challenges in rapidly separating informative evidence from research that may be less useful or misleading due to poor methodology, inadequate reporting, or both. To tackle this barrier to informed decision-making, we launched CovidReview.ca, an open-access online platform that screens all available COVID-19 research to determine higher quality publications classified based on the Levels of Evidence Pyramid [7]. CovidReview is continually updated to ensure that new emerging evidence that may inform decision-making is included. All papers are organized by type of research, specialty of interest, journal, and type of publication.

This paper aims to explore the trends in published COVID-19 research worldwide since its outbreak in December 2019, specifically assessing the number of publications over time categorized by type of research. We hypothesized that the most common type of publication would be narrative review/expert opinion, but that the number of randomized controlled trials (RCTs), systematic reviews, and meta-analyses would increase over time. This study is unique as it tracks the direction, rate of publication, and evolution of research for an entirely novel pandemic disease through the creation and utilization of a novel online database.

## Materials and methods

Eligibility

This project was designed to be a scoping review of all COVID-19-related publications; therefore, all articles captured by our search strategy were reviewed. The primary objective of this project was to screen all published COVID-19 literature based on study design using the Levels of Evidence Pyramid [7] (further discussed in the Article Categorization section) and medical specialty in order to create a curated online database of medical research (CovidReview.ca) valuable for healthcare professionals. The secondary objective of this project - and the primary objective of this article - is to analyze trends in the type of literature related to COVID-19 published over time. Although our scoping review continues (as of July 2020) to screen all publications on an ongoing basis, we included in our analysis publications indexed on PubMed until an arbitrary end date of May 10, 2020.

Search strategy

The literature search strategy included "COVID-19”, “severe acute respiratory syndrome coronavirus 2”, “coronavirus 19”, “SARS-COV-2”, and “2019-nCoV” to account for variations in COVID-19 nomenclature. PubMed was the sole database used for the purposes of this project. The initial search was conducted on PubMed March 28, 2020 (n=1,890 articles). We repeated the search periodically to capture newly published articles on April 8, 2020 (N=3,288), April 15, 2020 (N=4,298), April 29, 2020 (N=7,649), May 6, 2020 (N=9,163), May 13, 2020 (N=11,464), May 20, 2020 (N=14,360), and May 27, 2020 (N=16,128).

Article selection

Articles were uploaded into DistillerSR (Evidence Partners, Ottawa, Canada). Nine independent reviewers (B.J., J.H., H.K., D.L., M.G., M.A., J.H., J.J., S.M.) completed title and abstract (i.e., level 1) screening using a predetermined screening form. Each article was reviewed by a minimum of two reviewers to assess the type of research, medical specialty of relevance, type of publication, and inclusion or exclusion to the CovidReview.ca repository. Discrepancies were resolved between the original reviewers through consensus discussion or, in rare cases, a third reviewer. Inter-rater reliability using Cohen’s kappa was calculated by ((observed % of agreement) - (expected % of agreement) / 1 - (expected % of agreement)).

Article categorization

On the predetermined screening form, reviewers were asked to identify the type of research, relevant medical specialties, and type of publication (guidance document, report of intervention/treatment, etc.) for each paper. Articles were divided into “common” and “uncommon” specialties for the purposes of categorization. Common specialties (specialties under which common signs, symptoms, and complications of COVID-19 infection would normally be observed or managed) included anesthesia, emergency medicine, family medicine, intensive care unit, immunology, infectious disease, palliative care, radiology, and respirology. Uncommon specialties (specialties under which common signs, symptoms, and complications of COVID-19 infection would not typically be observed or managed) included cardiology, dermatology, ENT, gastroenterology, geriatrics, hematology, nephrology, neurology, obstetrics & gynecology, oncology, ophthalmology, pathology, pediatrics, physiatry & rehabilitation medicine, psychiatry, rheumatology, surgery, and urology.

Inclusion/exclusion criteria for randomized controlled trials, observational studies, meta-analyses/systematic reviews, and narrative reviews

For inclusion to the website, studies must have been clinically relevant for a wide variety of clinical settings. This could include research relevant to the diagnosis, prognosis, treatment, etc. of COVID-19. Studies that had unique findings that enhanced the overall clinical understanding of COVID-19 were included. Articles regarding topics related to uncommon specialties or important public health concerns were also included. Studies that were solely relevant to specific cities/countries were excluded.

Inclusion/exclusion criteria for case reports/series

A case report or case series was included if it described a unique presentation (i.e., symptoms not commonly reported) of COVID-19. This encompassed rare, uncommon, or atypical presentation or complications of COVID-19 infection, or unique or rare comorbidities associated with COVID-19 infection. Case reports/series were also included if they described the management of concurrent COVID-19 infection in patients with a condition classified under an uncommon specialty or if the paper reported on unexpected interactions between the condition and concurrent COVID-19 infection in regard to presentation, diagnosis, or treatment, or unique or novel diagnostic measures or interventions for COVID-19 in patients with the condition. Case series in common specialties describing typical presentations of COVID-19 (typical symptoms included cough, fever, fatigue, sore throat) were included if they had >10 patients.

Inclusion/exclusion criteria for animal/lab studies

Animal or laboratory studies were included if they presented unique findings that could be directly applied to clinical management or add to the overall understanding of COVID-19 symptoms and progression, including diagnostic tools and interventions (i.e. pharmacotherapy). Reports that discussed factors contributing to our understanding and treatment of the immune response to COVID-19 were included. Additional inclusion criteria included results that provided direct evidence regarding the efficacy of specific diagnostics or treatments. Articles that solely suggested avenues for investigating potential therapies, or articles that were only applicable to animal species, were excluded.

Inclusion/exclusion criteria for guidance documents

Detailed and objective evidence-based guidelines that were generalizable and applicable to a wide array of COVID-19-related clinical scenarios were included. These papers generally described guidelines related to direct treatment of COVID-19, protective measures to prevent nosocomial COVID-19 infection among patients and staff in the clinical setting, or management of a wide number of clinical scenarios. Expert-informed guidelines without a basis in direct research or evidence were also included if the guidelines were based on the recommendation of more than five experts (authors) or were based on experiences of clinicians from multiple centers. Guidelines that covered a wide array of common clinical scenarios directly related to the management of patients with COVID-19 or protection from COVID-19 infection were included. Papers that outlined measures that were only applicable to a specific site, described subjective experiences of clinicians, or did not specify any objective recommendations that were more widely applicable to clinicians globally were excluded.

Metadata processing and supplementation

All fully reviewed studies were downloaded in a bulk reference format from DistillerSR. Article metadata, particularly date of publication, were inconsistent across journals and recency of publication. To ensure a standardization of date metadata across all studies, Extensible Markup Language (XML) metadata were downloaded for all references from PubMed. PubMed index dates were extracted and used as surrogate publication dates. Metadata generated by two or more reviewers such as specialty of relevance were automatically combined, and the type of study was checked to ensure inter-reviewer consistency. As well, metadata were parsed to allow precise and fast searching by keywords, individual author names, ISO journal abbreviations, type of study, and specialties of relevance.

Web-tool development

Processed references for studies that passed screening are available online at https://www.covidreview.ca, which hosts an R Shiny application using Shiny Server [8,9]. Data from our screening are reviewed for errors and integrated manually into the database every two to three days (J.H.). Additional detail regarding the nature and development of the software is beyond the scope of this article; our data and source code are available at https://www.github.com/jzpero/covidreview.

## Results

**Figure 1:**
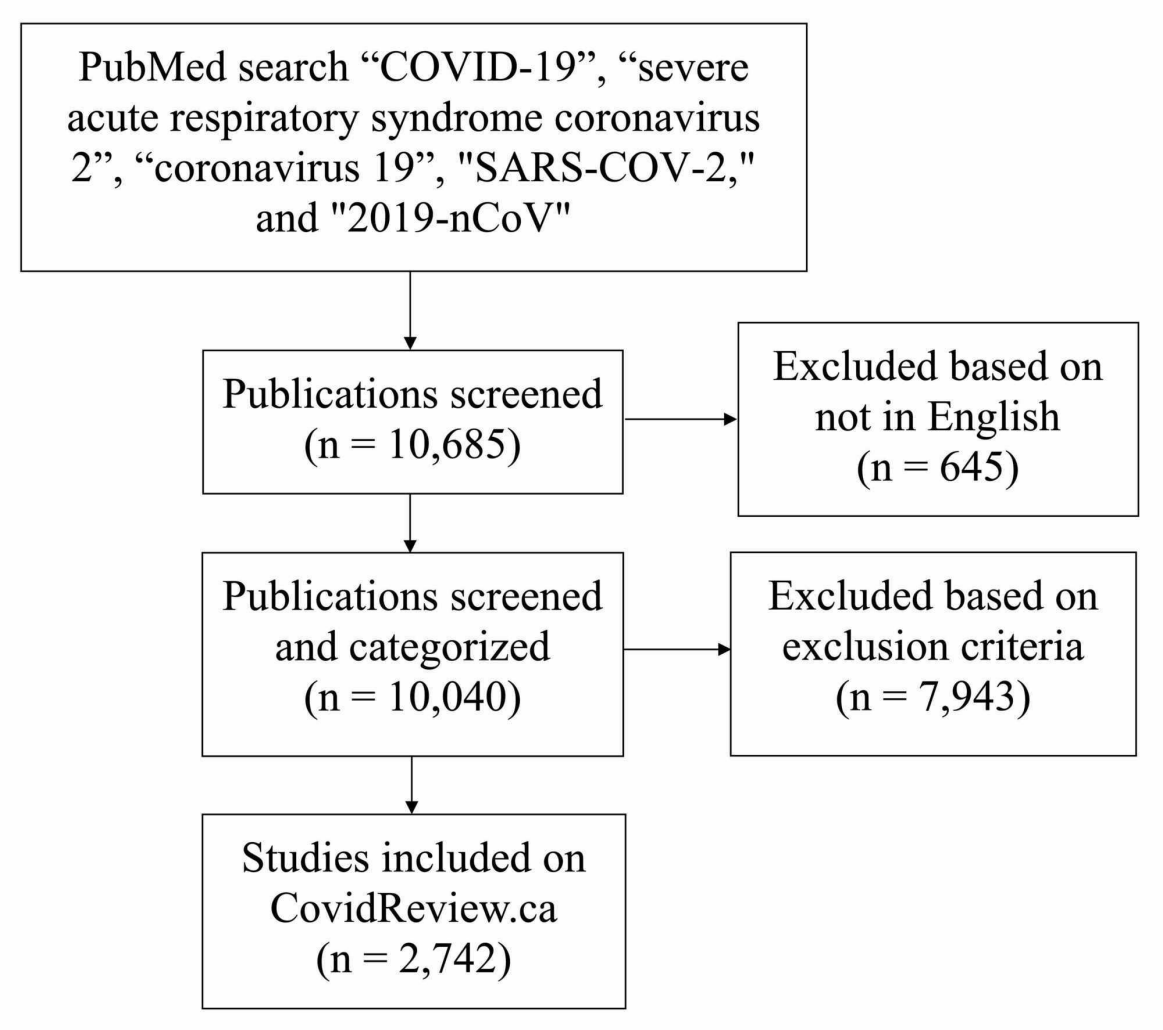
Search strategy and categorization process

The first SARS-CoV-2 (severe acute respiratory syndrome coronavirus 2) related references captured in our searches (in J Travel Med and Science) were indexed in PubMed on January 17, 2020. We extended our period of analysis for this paper from this date onward until May 10, 2020. In this time period, the total number of papers that met our search criteria was 10,685, of which 645 were excluded because they were not in the English language. Overall, 2,742 papers were included and made available to search on CovidReview.ca, with 7,943 papers excluded based on methods outlined previously (Figure 1).

**Figure 2:**
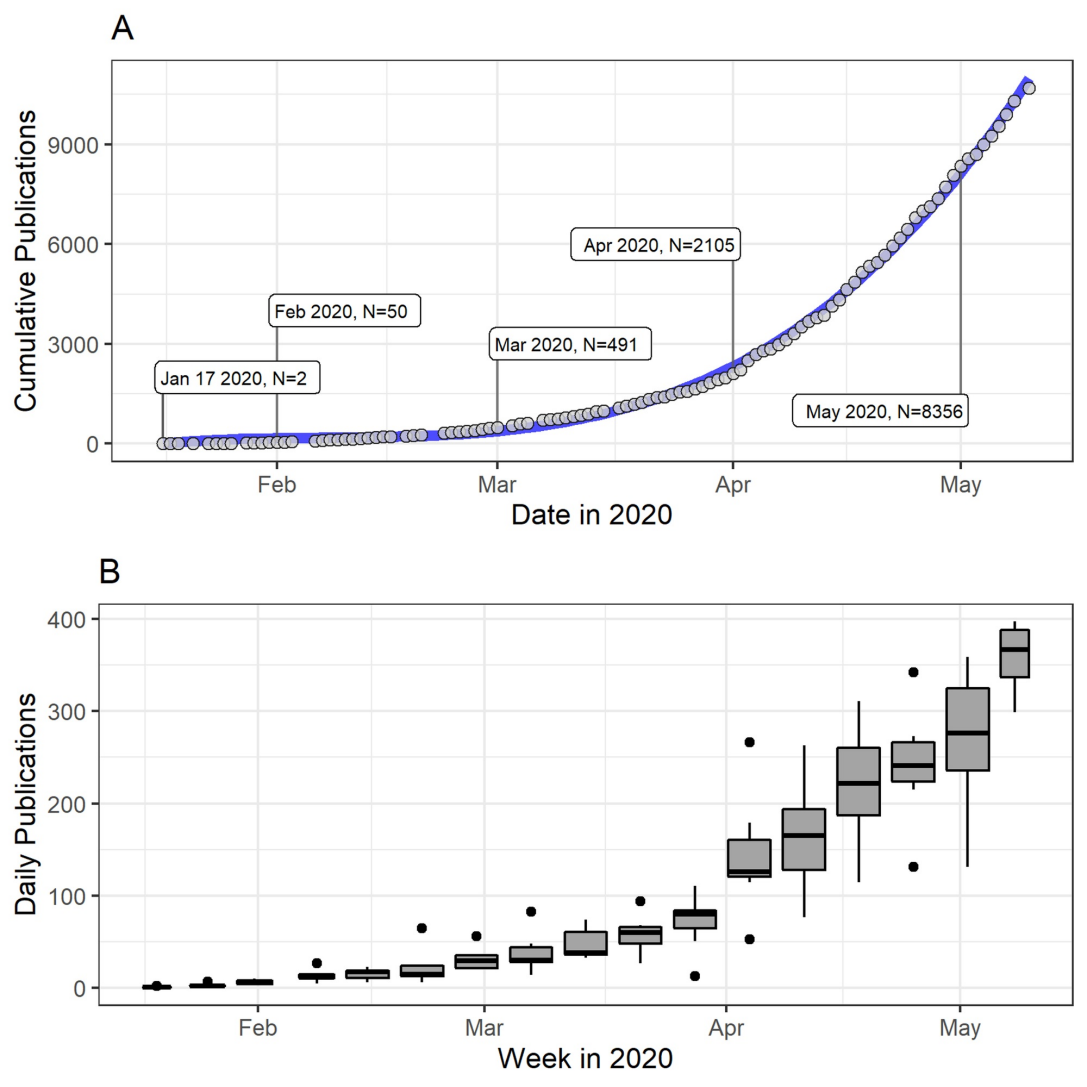
Number of COVID-19 references indexed in PubMed during early 2020 (A) and daily publications in each week of early 2020 (B). Data shown is from January 17, 2020, to May 10, 2020, inclusive. Linear regression produced an estimated line of best fit (blue) y=0.014x^3^ - 0.893x^2^ + 22.3x (R2 = 0.9993), where x is days. Weeks are defined as consecutive groups of seven days starting from January 1, 2020, onward.

The volume of publications captured over time increased dramatically over 16 weeks, with a third-order polynomial estimated line of best fit indicating that the number of publications increased significantly in the latter weeks of analysis compared to the former weeks, suggesting that publication rates will continue to rapidly increase over time (Figure 2A). In the final week of analysis (May 6, 2020, to May 10, 2020), mean daily publication rate was 358 per day, whereas in the first week of analysis (January 17, 2020, to January 21, 2020), it was only 1 per day (Figure 2B).

**Figure 3:**
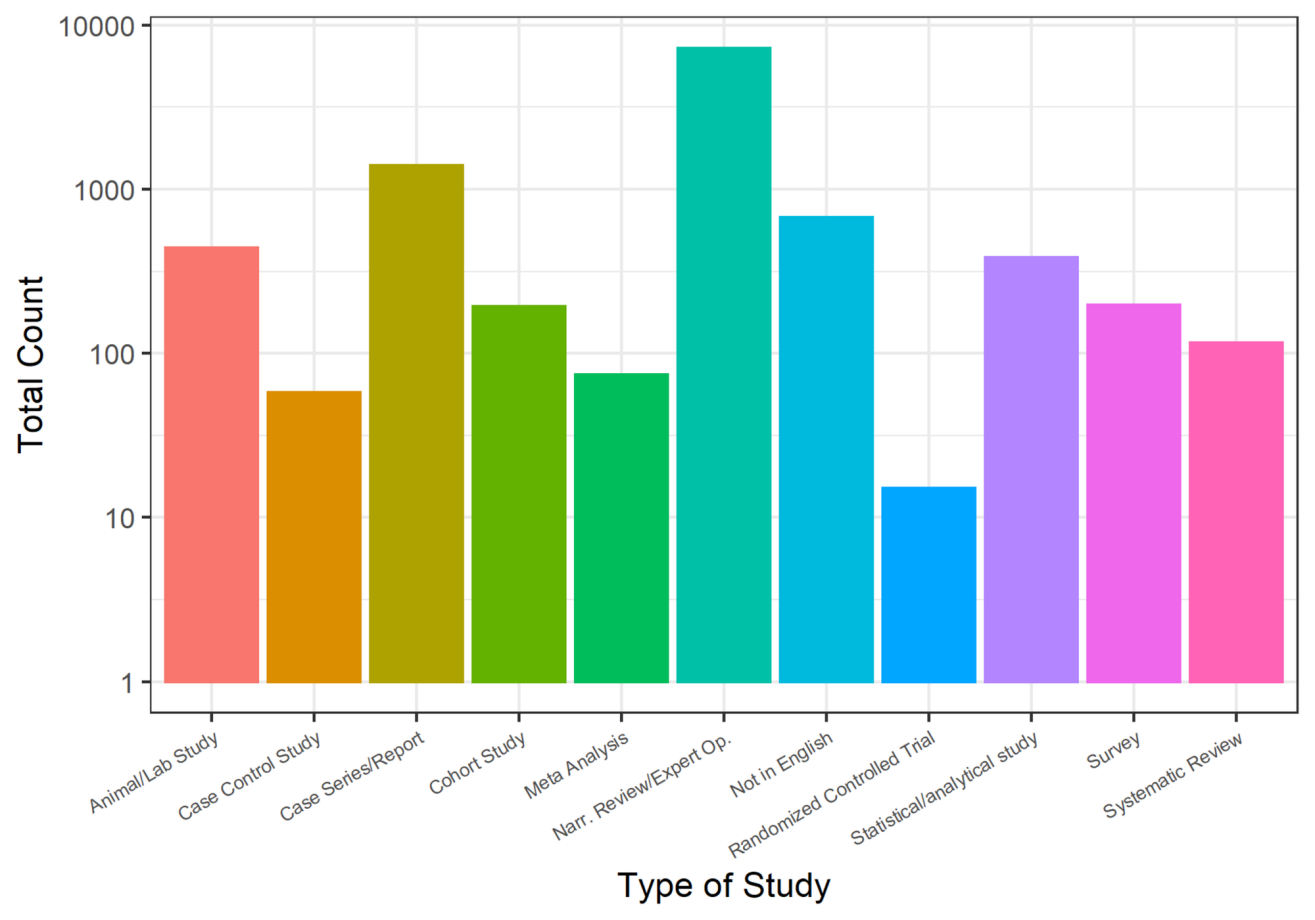
Types of COVID-19-related studies indexed in PubMed from Jan 17, 2020, to May 10, 2020 Narr. Rev/Expert Op., narrative review/expert opinion

**Figure 4:**
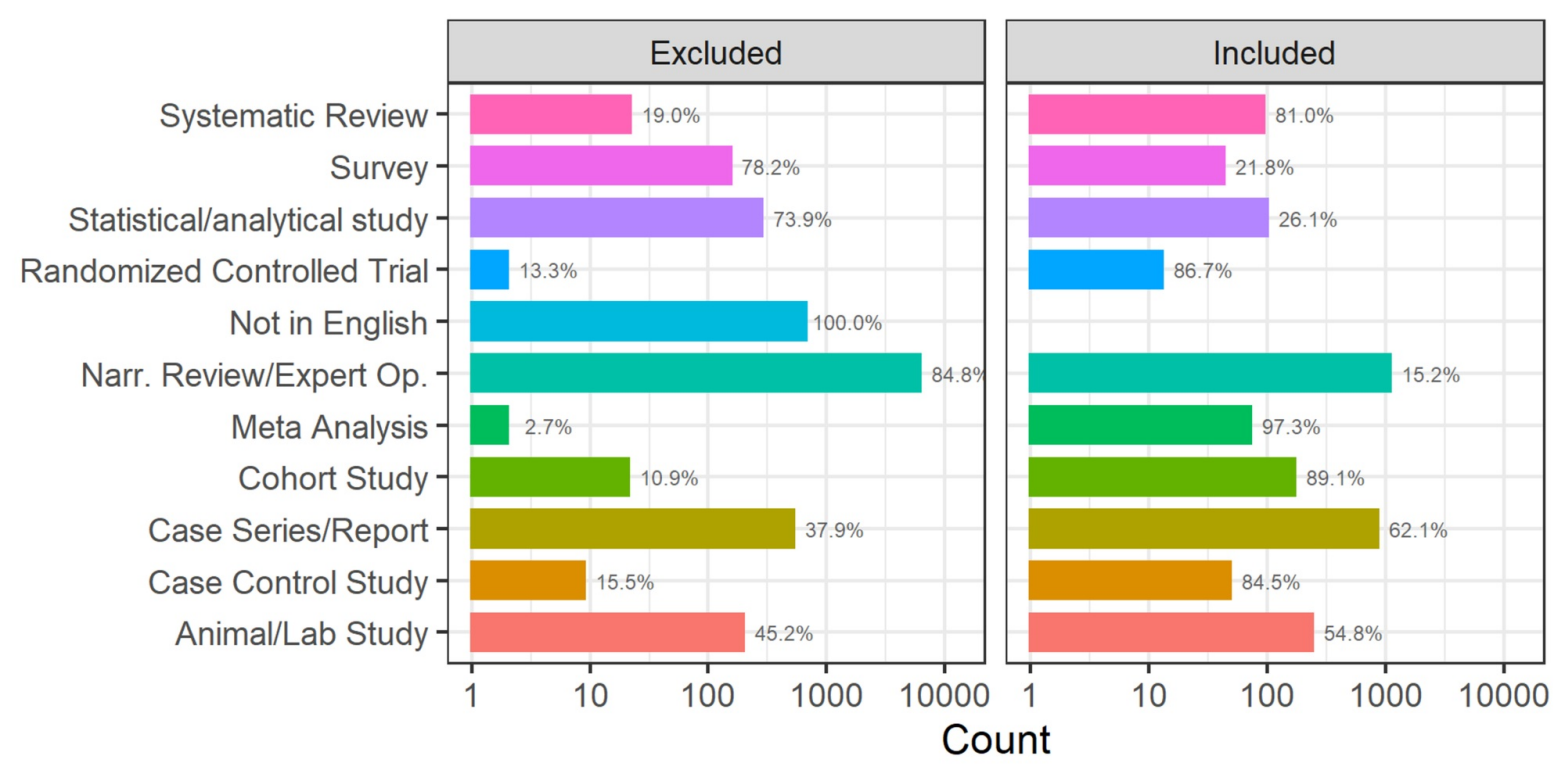
Studies by type and inclusion status from January 17, 2020, to May 10, 2020 Narr. Rev/Expert Op., narrative review/expert opinion

When measured at our study end date (May 10, 2020), the total literature comprised 74 meta-analyses, 116 systematic reviews, 15 RCTs, 58 case-control studies, 192 cohort studies, 1,385 case report or case series, 7,183 narrative reviews or expert opinion commentaries, 437 animal or laboratory studies, and 197 surveys (Figure 3). Included versus excluded study data can be found in Figure 4.

Narrative reviews and expert opinion papers had the highest exclusion rate as compared to RCTs, systematic reviews, and meta-analyses, which had the highest rate of inclusion per total papers published (Figure 4). Cohen’s kappa determining the degree of inter-rater reliability was 0.61.

**Figure 5:**
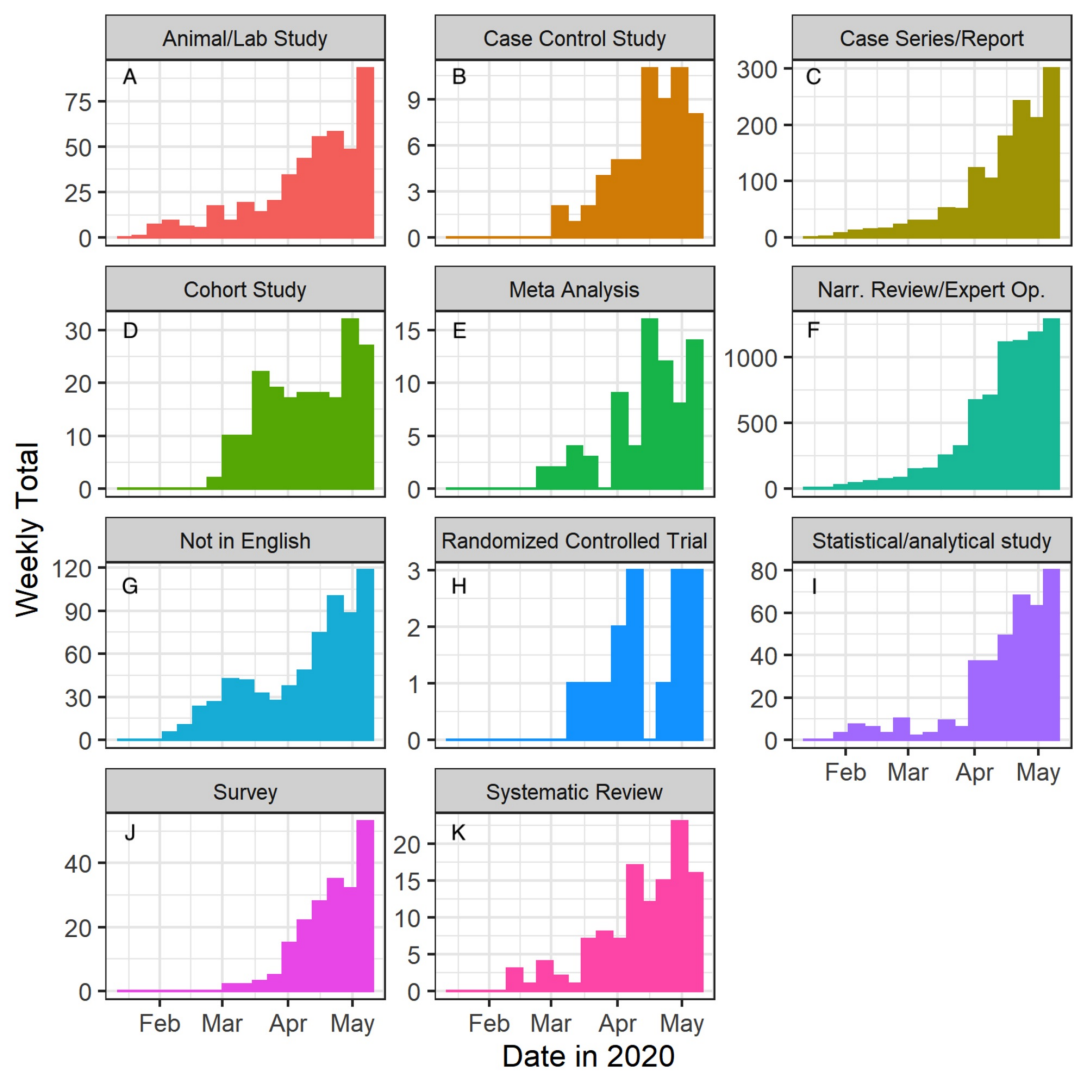
Types of studies published per week during early 2020 Narr. Rev/Expert Op., narrative review/expert opinion

**Figure 6:**
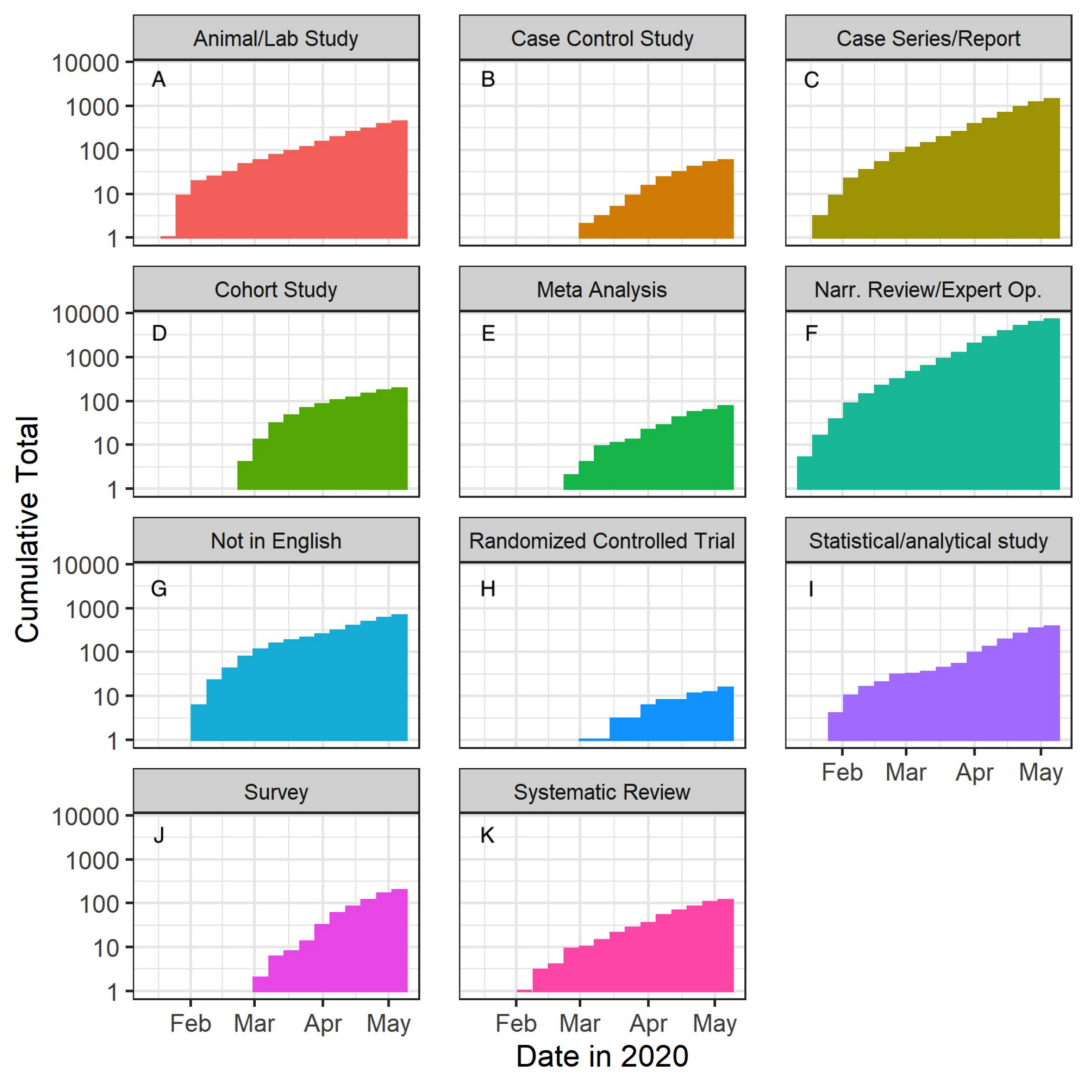
Cumulative studies sorted by type over time Narr. Rev/Expert Op., narrative review/expert opinion

The absolute number of studies published per week, in each category, also generally trended upward (Figure 5). The most published type of study over the 16 weeks were narrative review/expert opinion papers (N=7,183; Figures 5F, 6F) followed by case series/reports (N=1,385; Figures 5C, 6C). Meta-analyses (N=74; Figures 5E, 6E), systematic reviews (N=116; Figures 5K, 6K), and RCTs (N=15; Figures 5H, 6H) remained the least published types of studies throughout.

## Discussion

COVID-19 has inarguably become a primary focus of research facilities and medical institutions since being discovered in December of 2019. Massive efforts have been made to redeploy funding and research personnel in order to publish critical data in the fight against this global pandemic [10]. As outlined by Chahrour et al, [11], access to information and the speed by which the medical community acquires published data is an essential first step in the defense against a disease. The publication of almost real-time data is only possible in the form of case reports, observational studies, and narrative reviews, which can inevitably be distributed at a faster rate than RCT’s, meta-analyses, and systematic reviews. Over time, however, higher quality publications become invaluable for the long-term treatment and management of a COVID-19. The added functionality of organizing publications based on study type and specialty of medicine allowed clinicians to sift through information most relevant to their practice or research. Although this tool was initially designed as a resource for clinicians and scientists to quickly sift through COVID-related research that met our baseline criteria, the creation of this data pool has also allowed us to decipher and analyze the trends in research type and inclusion versus exclusion over time.

The surge in research over time is likely due to several factors. First, as the pandemic progressed globally and began to reach more centers in additional countries, new data were made available for publication. Secondly, primary research, particularly experimental studies, requires time to implement and analyze. Due to the novelty of COVID-19, it was to be expected that studies requiring careful planning and execution, such as RCTs, would require more time to be implemented and thus be published later on in the pandemic.

Although there was a general upward trend in the overall number of publications related to COVID-19 over time, what differed significantly was the volume of each specific study type. Unsurprisingly, narrative reviews were the most highly published study type, with publications in the thousands even in the first few weeks of analysis (Figure 5F). Case reports and case series were also highly published in the first few weeks of analysis (Figure 5C). In contrast, RCTs were produced at the lowest rate and number, with a maximum of 13 RCTs published in any given week (Figure 5H). This is inevitable considering the length of time, effort, and rigor needed to publish a study such as an RCT [12]. Increases in systematic reviews and meta-analyses were observed over time, consistent with both increasing numbers of total publications and the aggregative nature of secondary research.

Another interesting observation that emerged from our analysis was the number of each study type that was included for upload to CovidReview.ca. Higher quality papers based on the Levels of Evidence Pyramid [7] were included more often than excluded (Figure 3). Using the pyramid as our metric, research more likely to be included encompassed RCTs (86.7% included), systematic reviews (81.03% included), meta-analyses (97.3% included), cohort studies (89.18% included), and case-control studies (84.5% included). Case series/reports (62.4% included) and animal/laboratory studies (54.9% included) were also included more often than excluded despite being lower on the evidence pyramid. Comparatively, narrative reviews (15.4% included), statistical modelling studies (26.0% included), and surveys (21.8% included) were more often excluded than included. The inclusion rates reflect the importance of publishing studies higher on the evidence pyramid to provide guidance to frontline clinicians and researchers.

Other literature has similarly found a rapid increase in production of COVID-19 research since January 2020 [11,13]. These studies identified that the majority of papers are coming from areas highly impacted by COVID-19, such as China, the United States, and Europe [11,14]. In addition, much of the current research focuses on themes and topics discussed in published papers. Zhang and Shaw [15] and Liu et al. [16] identified that a large amount of research has been focused on virology, epidemiology, and immunology. Liu et al. [16] also analyzed study-type trends and found that the majority of publications were opinion pieces, editorials, and news. This finding is congruent with the high output of narrative reviews and expert opinions seen in our study, which encompasses these subtypes of publications. Additionally, Chahrour et al. [11] and Liu et al. [16] reported a lack of RCTs, which was also affirmed by our study. Overall, the findings of our study, and others, collectively suggest the need to increase production of high-quality research, such as RCTs, systematic reviews, and meta-analyses, to better understand COVID-19 and explore potential interventions and treatments.

There are some limitations to our study. Primarily, the inclusion and exclusion criteria used to upload publications to CovidReview.ca may have been implemented slightly differently between reviewers. For example, the inclusion criteria warranted the paper have had unique or significant findings. What a reviewer believed to be a unique finding may have differed based on a reviewer’s experience or other biases, such as the sample of COVID-19 papers they had reviewed in the past. In addition, we only assessed articles that were published in English. Lastly, we did not include papers that were not open or institutional access, which also constrained our pool of articles. This was done to maintain the functionality of our website; therefore, all articles screened and published were open access or accessible by those with institutional access.

## Conclusions

During the 16-week span of our study (January 17, 2020, through May 10, 2020), the world witnessed a surge in research focused on COVID-19. As our data demonstrates, the majority of studies published in the literature thus far have been narrative reviews and case studies or case series, with a slow rise in RCTs, systematic reviews, and meta-analyses. It is clear from our analysis that in an effort to quickly provide the world with up-to-date information, the highest volume of primary research in early 2020 consisted of observational studies. Due to the novelty of COVID-19, a gap in knowledge currently exists around the treatment, prevention, and long-term sequelae of the disease. This paper highlights the sheer volume of COVID-19 research being published, which is impractical for practitioners to sift through. It is important for journals to recognize and filter studies based on design and applicability. Continued research which falls higher on the evidence pyramid and is more applicable to clinical settings is warranted.

## Appendices

**Table 1:** Summary of data

Type of Study	Total Included	Total Excluded	Overall Total	Inclusion Rate
Narrative Review/Expert Opinion	1099	6084	7183	15.30%
Case Series/Report	861	524	1385	62.17%
Animal or Laboratory Study	240	197	437	54.92%
Cohort Studies	171	21	192	89.06%
Statistical Modelling/Analytical Study	100	283	383	26.11%
Systematic Review	94	22	116	81.03%
Meta-Analysis	72	2	74	97.30%
Case-Control Studies	49	9	58	84.48%
Survey	43	154	197	21.83%
Randomized Controlled Trial	13	2	15	86.67%
Not in English	0	645	645	0.00%
Total	2742	7943	10685	

**Table 2:** Cumulative included studies by type and week of 2020 during the study period (January 17, 2020, to May 10, 2020)

Types of Included Studies, by Week (Cumulative)											
Week of 2020	Animal or Laboratory Study	Case-Control Studies	Case Series/Report	Cohort Studies	Meta-Analysis	Narrative Review/Expert Opinion	Not in English	Randomized Controlled Trial	Statistical Modelling/Analytical Study	Survey	Systematic Review
4	1	0	2	0	0	0	0	0	0	0	0
5	7	0	5	0	0	0	0	0	1	0	0
6	14	0	9	0	0	11	0	0	5	0	1
7	18	0	18	0	0	14	0	0	7	0	3
8	21	0	28	0	0	23	0	0	9	0	4
9	29	0	44	4	2	35	0	0	13	0	9
10	33	2	58	13	4	78	0	1	15	0	10
11	40	3	65	30	8	113	0	1	16	2	14
12	47	4	86	44	10	155	0	3	18	3	20
13	54	8	131	59	12	255	0	3	20	8	26
14	82	14	205	74	21	442	0	5	35	14	31
15	113	21	279	88	27	568	0	7	46	19	44
16	138	26	385	103	40	687	0	7	55	24	56
17	163	34	539	123	52	804	0	10	65	32	68
18	206	44	701	149	59	998	0	11	87	37	86
19	240	49	861	171	72	1099	0	13	100	43	94
Total Included		2742									

**Table 3:** Cumulative excluded studies by type and week of 2020 during the study period (January 17, 2020, to May 10, 2020)

Types of Excluded Studies, by Week (Cumulative)											
Week of 2020	Animal or Laboratory Study	Case-Control Studies	Case Series/Report	Cohort Studies	Meta-Analysis	Narrative Review/Expert Opinion	Not in English	Randomized Controlled Trial	Statistical Modelling/Analytical Study	Survey	Systematic Review
3	0	0	0	0	0	5	0	0	0	0	0
4	0	0	1	0	0	16	0	0	0	0	0
5	2	0	4	0	0	37	0	0	3	0	0
6	5	0	13	0	0	72	6	0	5	0	0
7	6	0	15	0	0	121	22	0	9	0	0
8	9	0	23	0	0	186	42	0	11	0	0
9	17	0	38	0	0	262	77	0	17	0	0
10	24	0	50	0	0	361	113	0	17	2	0
11	34	0	74	1	1	489	155	0	19	4	0
12	45	1	102	3	1	729	176	0	25	5	1
13	58	1	116	8	1	974	207	0	32	5	1
14	66	1	168	10	1	1495	239	1	61	17	4
15	78	2	215	14	1	2177	300	1	85	39	9
16	113	5	298	16	2	3139	382	1	131	58	10
17	140	6	385	19	2	4167	468	1	191	86	14
18	172	7	468	21	2	5237	571	1	249	129	20
19	197	9	524	21	2	6084	645	2	283	154	22
Total Excluded		7943									

**Table 4:** Most represented journals for COVID-19 literature in early 2020

Rank	Overall (% of Total)	Included (% of Included)	Excluded (% of Excluded)
1	BMJ (17.36)	J Med Virol (12.54)	BMJ (18.60)
2	J Med Virol (12.20)	J Infect (6.63)	Lancet (8.05)
3	Lancet (8.34)	Clin Infect Dis (6.51)	J Med Virol (7.46)
4	N Engl J Med (7.19)	N Engl J Med (4.26)	Nature (7.41)
5	Nature (7.09)	Int J Infect Dis (3.43)	N Engl J Med (5.95)
6	J Infect (5.47)	Radiology (3.31)	Science (5.25)
7	Science (5.32)	JAMA (3.20)	Infect Control Hosp Epidemiol (4.43)
8	Clin Infect Dis (5.16)	Head Neck (3.08)	Travel Med Infect Dis (4.37)
9	JAMA (5.01)	Am J Transplant (2.60)	JAMA (4.02)
10	Infect Control Hosp Epidemiol (4.64)	J Eur Acad Dermatol Venereol (2.60)	J Am Acad Dermatol (3.62)
11	Travel Med Infect Dis (4.59)	Lancet (2.60)	Lancet Infect Dis (3.56)
12	Lancet Infect Dis (4.17)	Intensive Care Med (2.49)	Rev Med Suisse (3.21)
13	Int J Infect Dis (4.12)	Br J Haematol (2.37)	Sci Total Environ (2.97)
14	J Am Acad Dermatol (3.65)	Crit Care (2.25)	Int J Infect Dis (2.92)
15	Head Neck (3.18)	Eur Respir J (2.25)	Int J Environ Res Public Health (2.86)
16	J Eur Acad Dermatol Venereol (3.18)	Lancet Infect Dis (2.25)	J Infect (2.86)
17	Ann Intern Med (2.97)	Emerg Microbes Infect (2.01)	Ann Intern Med (2.68)
18	Dermatol Ther (2.87)	Eur Radiol (2.01)	Euro Surveill (2.68)
19	Rev Med Suisse (2.87)	J Clin Virol (2.01)	Clin Infect Dis (2.57)
20	Euro Surveill (2.82)	J Thromb Haemost (2.01)	Dermatol Ther (2.51)

## References

[1] Guo Y, Cao Q, Hong Z (2020). The origin, transmission and clinical therapies on coronavirus disease 2019 (COVID-19) outbreak - an update on the status. Mil Med Res.

[2] (2020). Coronavirus Disease (COVID-19) Situation Report - 141. https://www.who.int/docs/default-source/coronaviruse/situation-reports/20200609-covid-19-sitrep-141.pdf?sfvrsn=72fa1b16_2.

[3] (2020). Coronavirus Disease (COVID-19) Pandemic. https://www.who.int/emergencies/diseases/novel-coronavirus-2019.

[4] (2020). Coronavirus Disease 2019. https://www.cdc.gov/coronavirus/2019-ncov/index.html.

[5] Homolak J, Kodvanj I, Virag D (2020). Preliminary analysis of COVID-19 academic information patterns: a call for open science in the times of closed borders. Scientometrics.

[6] Alexander PE, Debono VB, Mammen MJ (2020). COVID-19 coronavirus research has overall low methodological quality thus far: case in point for chloroquine/hydroxychloroquine. J Clin Epidemiol.

[7] Murad MH, Asi N, Alsawas M, Alahdab F (2016). New evidence pyramid. Evid Based Med.

[8] (2020). Shiny: Web Application Framework for R. https://CRAN.R-project.org/package=shiny.

[9] (2020). The R Project for Statistical Computing. https://www.R-project.org/.

[10] Haghani M, Bliemer MC (2020). COVID-19 pandemic and the unprecedented mobilisation of scholarly efforts prompted by a health crisis: scientometric comparisons across SARS, MERS and 2019-nCov literature [PREPRINT]. bioRxiv.

[11] Chahrour M, Assi S, Bejjani M, Nasrallah AA, Salhab H, Fares M, Khachfe HH (2020). A bibliometric analysis of COVID-19 research activity: a call for increased output. Cureus.

[12] Flecha OD, Douglas de Oliveira DW, Marques LS, Gonçalves PF (2016). A commentary on randomized clinical trials: how to produce them with a good level of evidence. Perspect Clin Res.

[13] Dehghanbanadaki H, Seif F, Vahidi Y, Razi F, Hashemi E, Khoshmirsafa M, Aazami H (2020). Bibliometric analysis of global scientific research on coronavirus (COVID-19). Med J Islam Repub Iran.

[14] Tran BX, Ha GH, Nguyen LH (2020). Studies of novel coronavirus disease 19 (COVID-19) pandemic: a global analysis of literature [PREPRINT]. medRxiv.

[15] Zhang H, Shaw R (2020). Identifying research trends and gaps in the context of COVID-19. Int J Env Res Pub He.

[16] Liu N, Chee ML, Niu C (2020). Coronavirus disease 2019 (COVID- 19): an evidence map of medical literature. BMC Med Res Methodol.

